# Sunburn as a Cause of Unexpected Neutrophilia in a Healthy Pregnant Woman

**DOI:** 10.1155/2018/8392127

**Published:** 2018-04-19

**Authors:** Robert Harper

**Affiliations:** North Devon District Hospital, Raleigh Park, Barnstaple EX31 4JB, UK

## Abstract

**Background:**

Neutrophilia has a broad differential diagnosis and represents a systemic response to an infection or other inflammatory pathologies.

**Case:**

A 31-year-old woman, Gravida 3, Para 2 at 28 weeks of gestation, presented to the day assessment unit following routine blood tests that showed an unexpected marked neutrophilia. The underlying cause of the neutrophilia was sunburn. The sunburn recovered and her neutrophil count spontaneously normalised.

**Conclusion:**

Clinicians can add sunburn to the broad differential diagnosis of neutrophilia.

## 1. Introduction

Neutrophilia refers to a higher than normal count of neutrophils in venous blood [1]. Neutrophils are phagocytes and a vital part of the acute inflammatory response to a range of stresses [1, 2]. Cytokines released during stress cause neutrophilia due to movement of neutrophils from the marginal pool into the circulating pool of blood and an increase in production of neutrophils from the bone marrow [2]. In clinical practice, neutrophilia is most commonly encountered in the setting of infection, but clinicians need to be aware of the varied aetiologies of neutrophilia when constructing a differential diagnosis.

## 2. Case Presentation

A 31-year-old, Caucasian, Gravida 3, Para 2 woman presented to the day assessment unit. She had had two previous uncomplicated normal deliveries and no previous medical or surgical conditions. Her 1st trimester screening had shown a low risk for chromosomal disorders and no infection with blood borne viruses. Her 2nd trimester ultrasound scan had shown a normally grown foetus with no anomalies seen. She was under midwife-led care and had a regular attender for her antenatal appointments. Her blood pressure and urinalysis were normal at all appointments.

At 28 weeks of gestation, she attended her antenatal clinic for blood tests as part of routine antenatal screening. A full blood count, taken a week previously, had been normal. On this occasion however, her total white cell count was 29.9 × 10^9^/L, consisting of a neutrophil count of 23.92 × 10^9^/L with concurrent basophilia and eosinophilia (Table 1). Her platelet count was also raised compared to a week previously. Her haemoglobin was 106 g/dL, with a mean corpuscular volume of 69.5 fL. She was asked to attend the day assessment unit at her secondary care centre the following day for a repeat blood test and further assessment.

The patient had been entirely well over the preceding weeks. She reported no symptoms of acute or chronic infection. She had no abdominal pain or vaginal discharge or bleeding. Foetal movements and cardiotocography were normal. She had not started any new medications in the preceding weeks. She confirmed that she had no known chronic inflammatory conditions, had not had any trauma recently, and felt under no particular mental stress.

On examination, she had a soft and nontender abdomen and an appropriately grown gravid uterus. Her chest was clear and her heart sounds normal. There was no evidence of deep vein thrombosis. On examination of her skin, erythematous patches and overlying desquamation were noted in sun-exposed areas, principally the shoulders. She had a skin phototype [3] of 2 (usually burns, rarely tans). Her blood pressure and urinalysis were normal. A repeat full blood count confirmed neutrophilia, basophilia, and eosinophilia. A blood film confirmed neutrophilia and left shift and markedly microcytic red cells, with no evidence of haematological malignancy.

The weather in the UK had been exceptionally hot and sunny over the preceding week. The UV index, a measure of the strength of sunburn-inducing UV radiation, had peaked at 7 in our town in the days before her presentation [4]. A UV index of 7 is unusual in the UK and causes a “high” level of UV exposure and risk of sunburn [3]. On further questioning, the patient confirmed she had spent an afternoon on the beach 4 days before her presentation and had neglected to protect her skin with clothes or sun cream, and in consequence she had been sunburnt. She felt that her skin was improving by the time of presentation.

The patient was given a provisional diagnosis of neutrophilia induced by sunburn and was managed conservatively. She was given sun safety advice and started on ferrous sulphate for possible iron deficiency anaemia. At telephone follow-up 1 week later, she continued to feel entirely well and her sunburn was settling. Follow-up full blood count showed normalisation of her blood tests, confirming the provisional diagnosis. Furthermore, the incidental finding of microcytic anaemia had been resolved by her course of oral iron.

## 3. Discussion

The differential diagnosis of neutrophilia is broad (Table 2). Of particular relevance to pregnancy are infections, preeclampsia, corticosteroid administration, and the stress response to labour and delivery. It should also be noted that the normal range of the neutrophil count is increased even in an entirely uncomplicated pregnancy [5]. A full history and examination are usually sufficient to elucidate the cause of neutrophilia; however further investigations that may be necessary include biochemistry, microbiological samples, and relevant imaging. If there is suspicion of haematological malignancy, blood films and bone marrow biopsy must be undertaken by the relevant specialists.

Sunburn is caused by ultraviolet B (UVB) light [6]. UVB light passing through keratinocytes can cause DNA damage by inducing adjacent pyrimidine bases to form pyrimidine dimers [7]. The reaction is common and is usually quickly repaired by nucleotide excision repair [8]. However, the DNA damage induces keratinocytes to produce high levels of proinflammatory cytokines, including IL-1, IL-6, TNF-alpha, and G-CSF [9]. G-CSF (granulocyte colony stimulating factor) specifically causes increased production and release of inflammatory cells such as neutrophils by the bone marrow. Neutrophils are then recruited to the site of tissue damage and contribute to inflammation and repair [10]. Localised inflammation following excessive UVB exposure is noted clinically as erythema and subsequent desquamation in sun-exposed areas in the days following exposure, known as “sunburn” [11–13].

Thermal burns are noted to cause neutrophilia in texts [1, 2], but sunburn, although a common cause of burns in Caucasian populations, is not specifically documented as a cause. However, it is clear that sunburn is the clinical manifestation of an acute inflammatory response in the skin that has numerous effects, including a neutrophilia.

In this case, clinical examination, taken with knowledge of the local context and the author's own experience of the fine weather of the preceding weeks, allowed a clinical diagnosis to be reached without unnecessary further investigations. Follow-up, showing spontaneous resolution of the neutrophilia, confirmed the diagnosis. Clinicians who include sunburn in their list of differential diagnoses may be able to avoid unnecessary and potentially expensive and dangerous testing and intervention. Finally, pregnant women, like all patients, should be advised to avoid excessive sun exposure to prevent sunburn and the long-term risks of skin cancer.

## Figures and Tables

**Table 1:** Serial blood result chart (normal ranges from 2nd trimester [5]).

	Normal range	13/06/2017	20/06/2017	21/06/2017	22/08/2017
Haemoglobin (g/L)	110–150	108	106	101	129
White cell count (×10^9^/L)	6–16	8.61	29.90	26.94	12.1
Platelets (×10^9^/L)	150–400	124	264	250	195
Neutrophils (×10^9^/L)	4–13	6.66	23.92	20.90	8.53
Lymphocytes (×10^9^/L)	1–3.6	0.75	2.09	1.99	1.91
Monocytes (×10^9^/L)	0.1–1.4	0.92	2.09	2.88	1.20
Eosinophils (×10^9^/L)	0–0.6	0.21	0.90	0.57	0.26

**Table 2:** Examples of causes of neutrophilia [1, 2].

Physiological	Pregnancy, labour and childbirth, and strenuous exercise
Acute infection	Bacterial, viral, parasitic, and fungal infections
Chronic infection and “surgical” conditions	Abscesses, osteomyelitis, appendicitis, and cholecystitis
Noninfectious inflammation	Gout, rheumatic fever, and asthma
Tissue trauma	Burns and myocardial infarction, postoperatively
Metabolic	Preeclampsia and diabetic ketoacidosis
Drugs and toxins	Corticosteroids, adrenaline, and poisons
Malignancy	Haematological malignancies and nonhaematological malignancies producing growth factors or cytokines
Other	Hereditary and idiopathic neutrophilia syndromes and convulsions
